# VeriPriv: a privacy preserving attribute verification protocol for decentralized authentication

**DOI:** 10.1007/s10586-026-06646-0

**Published:** 2026-10-22

**Authors:** Mohammed Alshawki, Yuping Yan, Fatemeh Stodt, Christoph Reich, Peter Ligeti

**Affiliations:** 1 Cyber Security and Cryptography Research Group, Department of Computer Algebra, ELTE Eotvos Lorand University, Budapest, Hungary; 2 School of Engineering, Westlake University, Hangzhou, People’s Republic of China; 3 Institute for Data Science, Cloud Computing and IT Security (IDACUS), Hochschule Furtwangen, Furtwangen im Schwarzwald, Baden-Württemberg Germany

**Keywords:** Decentralized authentication, Privacy preserving protocol, Attribute based encryption

## Abstract

Attribute-based verification becomes more and more popular compared with identity-based authentication from the privacy point of view. Users can be authenticated by pieces of attributes rather than the whole identity. Attribute-based anonymous credential systems enable a user to prove possession of a given set of attributes to a verifier, without revealing information about other attributes. However, the challenge lies in how to securely prove the possession of attributes. In our paper, we propose a decentralized zero-knowledge protocol for attribute verification. There are three attribute verification modes, namely *1-out-of-n*, *n-out-of-n*, and the *Combined Verification Mode*. The first two modes represent the "OR" and "AND" gates, while the Combined Verification Mode supports an OR of non-empty groups of AND attributes. We prove the security properties of the protocol in the security analysis. Meanwhile, we also outline some of the use-cases and applications of the proposed protocol, such as Internet of Things(IoT), federated learning and data validation.

## Introduction

With the third-party cloud storage and cloud computing service providers, and a significant increase of public privacy awareness, Attribute-based Encryption(ABE) technology is the preferred solution to data protection and access control problems. Comparing to the public key encryption systems, ABE reduces the encryption cost by converting the one-to-one encryption mode into one-to-many. Meanwhile, as a variant of identity-based encryption, ABE provides a higher privacy level by verifying the attributes of individuals instead of the whole identity.

Assume a university library with a free Internet service to the professors and students (both full time and exchanged) of that university. In this case, only the fact that a person is a professor or a student of the same university, whether full time or exchanged student, is sufficient to be able to use the Internet service. However, a traditional approach that uses the person’s identity card to authenticate and prove the professorship or studentship relationships reveals a lot more information than it was required, as the identity cards usually include other sensitive data of the persons. Same issues happen in traditional online authentication protocols. Additionally, one might not want to reveal her exact relationship (e.g. being a professor, a full time student, or an exchanged student), other than the fact that she holds one of the defined attributes that allows her to legitimately uses the Internet service. Attribute-based encryption, verification and authentications are effective and practical in the real scenarios.

However, a major privacy challenge is how to authenticate the various members of a system and their attributes yet manage and protect the privacy of individual digital identities. Considering the privacy concerns, attribute verification became more important in many applications. Moreover, assume an Internet of Things(IoT) auditing system [1] where involving in the auditing process requires that an entity having a predefined set of attributes. Considering the privacy requirements, an auditor in the system wants to only prove the fact that it has all the required defined attributes to be able to join the auditing process, without actually revealing any other information about those attributes or its other attributes that are not part of the auditing requirements. The digital verification of attributes for an entity is a confront, and currently there are not a lot of researches or results for this problem.

To reach the target of attribute verification, one of the approaches is the Attribute-based anonymous Credential(ABC) systems, which was first introduced by Chaum [2]. The core idea in such systems [3, 4], is to allow a user to obtain attributes from an attribute authority, then use it to access resources which is encrypted by access policies while keeping the used credentials during the authentication process anonymous. In ABC systems, there is the *issuers* for attribute assurance, and the *verifier* for attribute verification. However, some issues make the adoption of systems challenging. The main problem is the utilization of the centralized managing entity that manages the main system functionality. It requires various entities to rely on this trusted entity for parts of the system functionality is among those challenges. Once the central entity get compromised, the whole attributes issue and verification system will get crashed. This requirement can negatively affect the system’s scalability and security.

As we can see, ABC systems enable a user to prove possession of a given set of attributes to a verifier, without revealing information about other attributes. However, the challenge lies in how to securely verify attributes in a decentralized manner without relying on a central managing entity. This motivates the design of decentralized mechanisms that can prove possession of attributes while limiting the disclosed information. Recent anonymous-credential constructions continue to study selective attribute disclosure, efficient credential presentation, and formally defined anonymity and unforgeability, demonstrating the continuing need for rigorous privacy-preserving attribute-verification mechanisms [5, 6].

### Our contributions

To fill the gaps in this research, we propose a decentralized zero-knowledge protocol for attribute verification in this paper. A preliminary version of this paper was presented at the 32nd International Telecommunication Networks and Applications Conference (ITNAC) [7]. The preliminary version introduced the basic protocol construction, the Decentralized Attribute-based Encryption(DABE)-based challenge, and the *1-out-of-n* and *n-out-of-n* verification modes. This journal version extends that work by formalizing the construction using the Linear Secret Sharing Scheme(LSSS) representation of the underlying DABE scheme, providing a formal security model and proofs, expanding the implementation and evaluation, and extending the threat and application analysis. The main contributions of this paper are the following:

- Formalizing VeriPriv, the decentralized attribute-verification construction using the LSSS-based DABE formulation and extending the access-policy support with a *Combined Verification Mode*, in addition to the existing *1-out-of-n* and *n-out-of-n* modes.
- Providing formal security experiments and analysis for soundness, attribute privacy, honest-verifier zero knowledge, unlinkability, and untraceability of the VeriPriv protocol.
- Providing a concrete implementation and a preliminary computational evaluation of the protocol, together with an expanded threat assessment, updated related work, and additional application scenarios such as IoT, Federated Learning(FL), and data validation.

The structure of the paper is the following. In Sect. 2 we introduce some related works in the field of attribute verification. Section 3 focuses on the ABE algorithms and ABC primitive. VeriPriv, the main attribute verification protocol is explained in Sect. 4 and analysed in Sect. 5. We point out the use-cases of the VeriPriv in Sect. 6. Finally, Sect. 7 includes our conclusions.

## Related work

Chaum [2] pioneered privacy-enhancing cryptographic protocols that minimize the amount of personal data disclosed, and the most effective approach is the anonymous credential systems. One of the most common seen anonymous credential systems is group signature based, such as the Camenisch-Lysyanskaya (CL) Idemix by IBM. The efficient CL signature scheme was first proposed by [8]. Based on the bilinear mapping, they propose this anonymous credential system with group signature. The core idea is to prove the equality of two committed values, get the signature based on the value (without revealing this value to the signer), and finally prove the knowledge of a signature on the committed value. This scheme is proved to be secure in the plain model and it enables selective disclosure and unlinkable multi-use.

Extending the pairing-based CL-anonymous credential system, [9] proposed the privacy-preserving predicate proof of attributes. In these protocols, one can prove the possession of all the multiple attributes (AND relation) and the possession of one of the attributes (OR relation). In this scheme, users are able to prove logic and comparison relations over attributes in an untraceable and unlinkable way. This proposal is very close to our research work, however, they applied CL-anonymous signature scheme while we use attribute-based encryption scheme. Meanwhile, our model is fully decentralized, compared to their centralized model.

In a similar direction, Camenisch et al. [10] introduced an RSA-based anonymous credential that encodes binary and finite-set values as prime numbers using zero-knowledge proofs. The scheme efficiently demonstrates the presence and absence of attributes by leveraging the divisibility and coprime properties. Furthermore, it also proves the validity of AND, OR, and NOT statements with value complexity. However, the strong RSA assumption comes with a high computational cost and low efficiency, while attribute proofs using pairing-based anonymous credentials require more research and effort. Other studies propose zero-knowledge proof techniques for demonstrating AND, OR, and NOT statements about attributes encoded as discrete logarithms. Belenkiy et al. [11] introduced a non-interactive proof system with P-signatures. It was the first signature non-interactive anonymous credential scheme, which does not rely on the random oracle model. From the attribute revocation point of view, Norio et al. [12] outlined a solution for efficient anonymous credential system based on strong Diffie-Hellman assumption. The security properties were proved in the aspects of perfect anonymity-unlinkability and computational unforgeability. Steuer et al. [13] introduced the identity attribute verification scheme in Windows CardSpace. It can compromise the semi-honest central identity manager and does not require information storage by third parties. Meanwhile, the researchers addressed the problem of linkability of digital identities in a privacy preserving protocol.

The research work by Wu et al. [14] presented a signature scheme to solve the problem of inefficient authentication of proving credential possession. Blömer and Bobolz [15] introduced a delegatable credential scheme that allows an attribute authority to delegate other users to issue a specific credential, hence a user in this model is able to authenticate herself using a derived credentials from other users. The model does not allow the verifier to trace the user where the credentials have been delegated from, but only the root authority. Closely to our work, Guo et al. [16] proposed an attribute proof scheme for smart devices. Their proposed model was based on random oracle pairing-based anonymous credential systems. It efficiently constructs anonymous credentials with cryptographical building blocks. Although the discussed results have important features, their implementation in our targeted applications discussed in Sect. 6 might be problematic, and can be implemented in specific applications where the existing of a single trusted entity, e.g. the root authority in [15] or [16], is allowed.

Recent studies have further investigated decentralized attribute management and privacy-preserving credential verification. Lu et al. [17] introduced multi-authority registered attribute-based encryption, in which independent key curators manage separate attribute sets and access policies can combine attributes from multiple authorities. Their pairing-based construction supports access policies represented by linear secret-sharing schemes, including monotone Boolean formulas. Wu et al. [18] proposed a decentralized multi-authority Ciphertext-Policy Attribute-based Encryption(CP-ABE) scheme for resource-constrained IoT environments, using outsourced computation to reduce the encryption and decryption burden on IoT devices. Chalkias et al. [19] introduced zero-knowledge authenticators for policy-private blockchain authentication, allowing users to prove authorization without revealing the underlying authentication policy. More recently, Kaviani and Setty [20] proposed Vega, a low-latency zero-knowledge proof system that enables users to prove statements about existing credentials without disclosing their remaining contents. Wang et al. [21] presented a decentralized multi-authority ABE scheme for eHealthcare that removes the central authority and reduces ciphertext size and encryption and decryption costs. These studies demonstrate continued interest in decentralized attribute management and zero-knowledge credential verification. However, they primarily address data-access control, credential presentation, or policy-private authorization, whereas our protocol focuses on decentralized verification of possession of attributes under *1-out-of-n*, *n-out-of-n*, and combined access policies.

## Attribute based encryption

Sahai and Waters proposed the concept of Fuzzy Identity Based Encryption(FIBE) [22] that led to the ABE algorithm. It was the first paper that proposes to use several attributes of a user to compose a (fuzzy) identity. Thus, it is also named as ABE. As a generalized concept to Identity Based Encryption(IBE) [23], ABE controls the access to the encrypted data by allowing not only a single user based on her identity, but a subset of users based on their attributes to being able to decrypt the ciphertext. Compared to the traditional public key cryptography, ABE provides a more flexible way of fine-grained access control by specifying the access policies. What is more, it converts the encryption and decryption mechanism from one-to-one to one-to-many by presenting a set of public keys for the available attributes, which is more suitable to the cloud computing environment scenario [24].

**Fig. 1:**
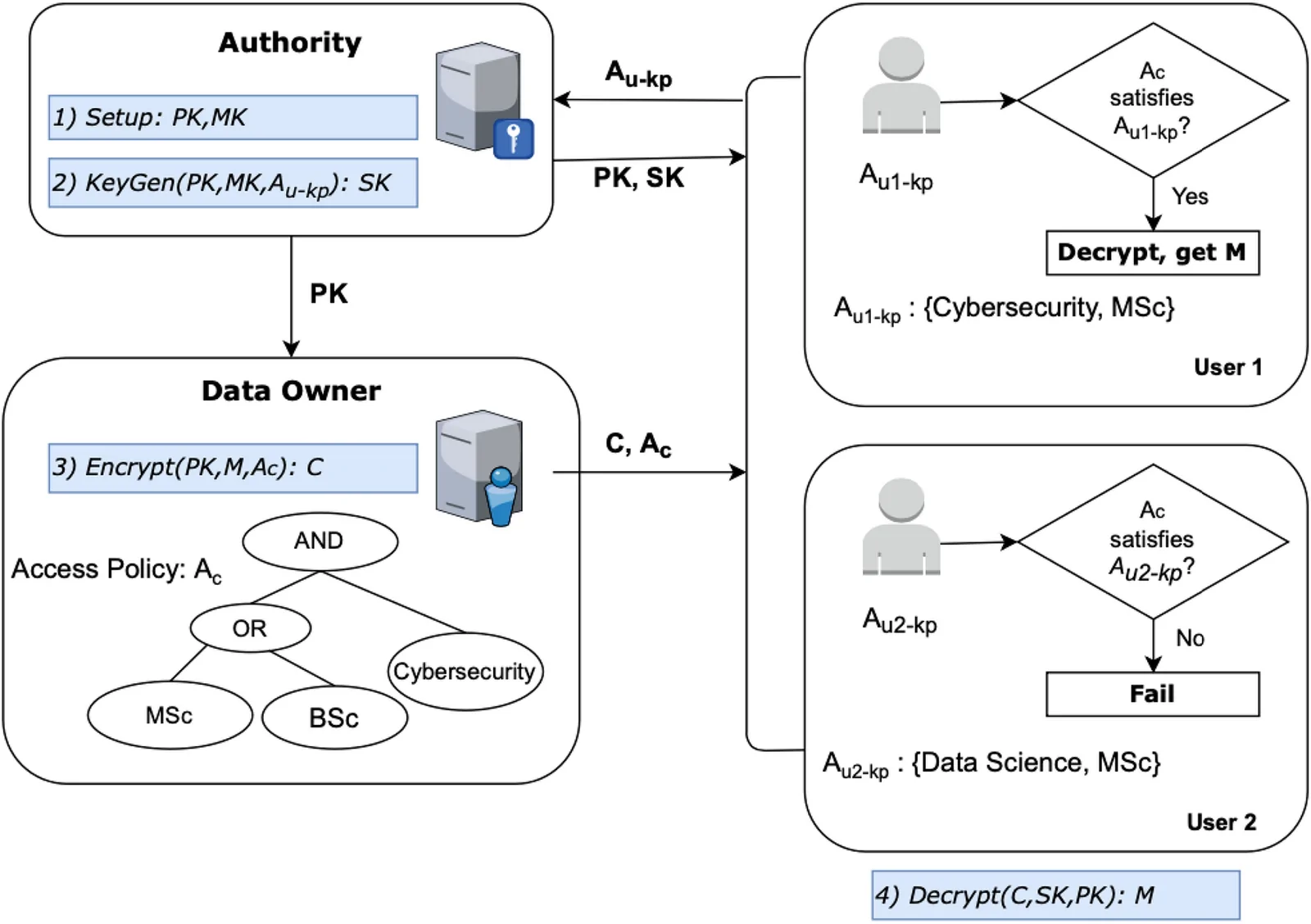
An overview of CP-ABE

There are two categories of ABE, which is divided the different access policies, namely Key-Policy Attribute-Based Encryption(KP-ABE) [25] and CP-ABE [26]. Both of these two types of ABE mechanisms can support the access control with attributes. However, in KP-ABE, the encryptor lists a set of descriptive attributes and sends these attributes to the users. Each private key is associated with an access structure that specifies which type of ciphertexts the key can decrypt. The user has private key, which is associated with an access structure that specifies which type of ciphertexts the key can decrypt. In this case, there is no authority check about data access because the users private keys are includes in access structure. In CP-ABE, ciphertext includes an access tree and users’ private keys includes user attributes. Users can verify its attributes with the access structure policy. Data access control is possible in this case since the access tree is in the ciphertext. This method is closer to traditional access control methods such as Role-based Access Control(RBAC). By comparing the differences, we can find out that CP-ABE provides a more flexible way for participants in our targeted application such as data validation network and federated learning by checking whether the attributes satisfy the access policies, and we adopt this technique in our model as well.

The overview of CP-ABE structure can be found in Fig. 1. The access policy $A_c$ is defined by the data owner. Users verify their own attributes $A_{u-kp}$ and check if it satisfies $A_c$ to decrypt the ciphertext. In this example, the access policy is the student should be a MSc or BSc and major in cybersecurity. The user1 meets the requirements and can decrypt the message, and the user2 can not decrypt as its attributes do not satisfy the access policy defined by the data owner.

However, the CP-ABE and KP-ABE has the same challenge of the centralized attribute authority, which decreases the privacy level and makes the system unpractical. To solve this challenge, the DABE was proposed, which is a decentralized variant of ABE. Recent studies have continued to investigate decentralized attribute-based access control. Wu et al. [18] proposed a decentralized multi-authority CP-ABE scheme with outsourced computation for resource-constrained IoT environments, while Shahraki et al. [27] presented a decentralized attribute-based access-control model in which individual domains retain their own policies and attribute authorities.

DABE [28] is also known as Multi-Authority Attribute-Based Encryption [29]. In this scheme, any party can be the authority by creating a public key and issuing private keys to different users that reflect their attributes. It reduces the computation cost and releases security issues of a single central authority in ABE, which makes the system more scalable. DABE protocol consists of five algorithms, $global\_setup()$, $attribute\_setup()$, $key\_generation()$, *encrypt*(), and *decrypt*(). We will briefly describe each of these algorithms. Before we describe the detailed protocol, we give some definitions as the background.

### Security assumptions

#### Definition 1

Let $P=\{P_1,P_2,...P_n\}$ be a set of *n* parties. An access structure is a collection *Γ* of non-empty subsets of *P*, i.e. $\Gamma \subseteq 2^{\{P_1,P_2,...P_n\}}$. Any $U\in \Gamma$ is called authorized set, and any $X\notin \Gamma$ is called unauthorized set. We say *Γ* is monotonic access structure if for any *M*, *N*, if $M \in \Gamma$ and *M ⊆ N*, then $N \in \Gamma$.

#### Definition 2

*(Bilinear pairing)* Let *G* and $G_T$ be two cyclic groups of prime order *p*, and let *g∈ G* be a generator. A pairing is a map $e:G \times G \rightarrow G_T$, which satisfies the following properties:

a. Non-degeneracy: $$\exists g \in G, e(g,g)\ne 1$$;

b. Bilinearity: $$\forall x,y \in Z_p, \forall h,\Theta \in G$$, $$e(h^x,\Theta ^y)=e(h,\Theta )^{xy}$$;

c. Computability: $\forall h,\Theta \in G$, there exists an efficient algorithm to compute *e(h,Θ )*.

Bilinear mapping is a type of function that can generate elements in a third linear space from elements in two other linear spaces, with all parameters being linear. Its role has become increasingly important in encryption structures, particularly in ABE and IBE. In many cases, the security of pairing-based protocols depends on variants of the Diffie Hellman problem that are closely related to bilinear mappings.

The protocol-level security proof does not directly reduce the challenge-response mechanism to the BDDH assumption. Instead, it reduces the security of the proposed protocol to the collusion resistance and chosen-plaintext security of the underlying DABE scheme. Therefore, the protocol inherits the hardness assumptions of the selected DABE instantiation rather than introducing an independent assumption for the challenge-response layer. If a DABE instantiation is proven secure under BDDH, the protocol-level reduction can be composed with that proof.

#### Definition 3

*(Bilinear Decisional Diffie–Hellman (BDDH) assumption)* Let *G* and $G_T$ be cyclic groups of prime order *p*, let *g* be a generator of *G*, and let $e:G\times G\rightarrow G_T$ be a bilinear pairing. For uniformly selected $a,b,c,z\in \mathbb {Z}_p$, no Probabilistic Polynomial-Time(PPT) algorithm can distinguish $(g,g^a,g^b,g^c,e(g,g)^{abc})$ from $(g,g^a,g^b,g^c,e(g,g)^z)$ with non-negligible advantage.

### DABE protocol

The DABE protocol that has been utilized as a building block in this paper is based on the decentralized attribute-based encryption scheme proposed by Lewko and Waters [28]. Unlike traditional ABE, it does not rely on a single central attribute authority. After a common global setup, independent attribute authorities can join the system, manage their own attributes, and issue attribute secret keys to users. The scheme consists of five main algorithms:

- $global\_setup()$: This algorithm takes the security parameter as input and generates the common global parameter used by all participants.
- $attribute\_setup()$: An attribute authority independently generates its private key and corresponding public key for the attributes it manages.
- $key\_generation()$: The attribute authority uses its private key and the global identifier of a user *u* to generate the corresponding attribute secret key.
- *enc*(): The encryption algorithm takes a message *M*, the global parameter, the public keys of the involved attribute authorities, and an access policy, and generates the ciphertext *C*. The access policy can include attributes managed by different independent authorities.
- *dec*(): The decryption algorithm takes the ciphertext *C*, the global parameter, and the user’s attribute secret keys. If the attributes associated with the same user identifier satisfy the access policy, the original message *M* can be recovered.

The detailed access-policy representation and reconstruction process follow the Lewko and Waters construction [28]. In this paper, we use this DABE scheme as the cryptographic foundation of the VeriPriv attribute-verification protocol.

## VeriPriv protocol for attribute verification

In this section, we describe VeriPriv, the proposed attribute verification protocol in detail.

### Participants

**Fig. 2:**
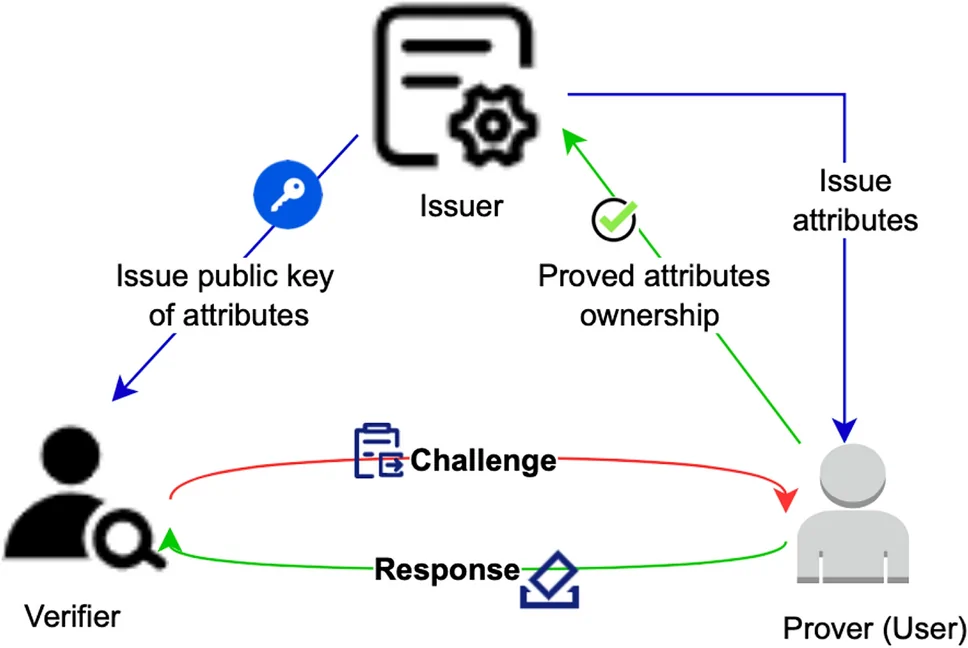
Types of participants and their relationship

The participants of VeriPriv can be divided into three types, the *issuer*, the *verifier* and the *prover* as shown in Fig. 2. The attribute authorities in previous ABC systems are the *issuers* in VeriPriv protocol. The roles of each participant are as follows:

1. **Issuer:** Issuer acts as the attribute authority. It is responsible for issuing the public key of an attribute to the verifier and issuing the corresponding attribute secret key to the prover (user).
2. **Verifier:** Verifier is responsible for verifying whether the attributes of the prover (user) satisfy the selected access policy. In the zero-knowledge setting, it challenges the prover for the proof.
3. **Prover (User):** Prover or the user is the entity that wants to prove possession of attributes. It uses the issued attribute secret keys to respond to the verifier’s challenge without revealing the secret keys or additional attributes.

After confirming that a user (later, the prover) has the claimed attribute, the corresponding issuer sends a secret key of the confirmed attribute to the user.

In the proposed model, we assume that the provers are independent nodes, each with a set of verifiable attributes known as the *Attribute Vector (AV)*. The attributes in each prover’s *AV* may come from different issuers, and the corresponding secret keys are issued by the corresponding issuers without cooperation from other issuers in the system. We also assume that the set of verifiable attributes related to each prover is *unforgeable*. This means that only the prover himself, and not any other node $v^* \in \mathcal {V} \setminus {v}$, can forge the related attributes of a given prover $v \in \mathcal {V}$ (the corresponding secret keys for the given attributes) with negligible probability only. By default, the attribute vector is private and known only to the prover node. However, there are certain applications, such as those described in [30], where the vector needs to be publicly published. In order to ensure the VeriPriv is sound and can prevent successful verification by malicious provers, it is important to maintain the privacy of the prover’s identity and attributes, apart from the fact that they possess the claimed attributes. This means that while the verifier needs to be able to confirm that the claimed attributes are present in the prover’s attribute vector, they should not gain any additional knowledge about the prover’s identity or other attributes. Despite the desire to minimize information disclosure, it is necessary for the proofs of attributes to guarantee some security requirements as follows.

### Formal security model

Let *λ* denote the security parameter, and let $\textsf{negl}(\lambda )$ denote a negligible function. We write $AV_u\models \Gamma$ when the attribute vector $AV_u$ satisfies the access policy *Γ*. The intended information disclosed by the protocol is limited to the value of the predicate $AV_u\models \Gamma$. In particular, the protocol does not attempt to hide whether the verification succeeds. The following analysis considers only information contained in the cryptographic protocol messages. Network identifiers, message timing, implementation side channels, and other external information are outside the scope of the model.

We assume that the underlying DABE construction is collusion resistant and secure against chosen-plaintext attacks under its stated security assumptions. In particular, an adversary that does not possess a set of attribute keys satisfying the challenge policy cannot distinguish the encrypted challenge key from an independently selected value except with negligible probability. We also assume that the symmetric-key encryption scheme *Π =(Gen,Enc,Dec)* is IND-CPA secure, and that the randomized public-key encryption scheme used for the prover’s response is IND-CPA secure.

The protocol-level reduction presented in Sect. 5 is composed with the established security and collusion-resistance properties of the selected DABE construction. If a different DABE instantiation is used, the corresponding hardness assumptions of that construction apply to the protocol in the same way.

#### Definition 4

*(Soundness experiment)* Let $\textsf{Exp}^{\textsf{snd}}_{\mathcal {P},\mathcal {A}}(\lambda )$ denote the following experiment between a challenger and a PPT adversary $\mathcal {A}$ acting as a malicious prover.

1. The adversary selects a target access policy $\Gamma ^*$.
2. The challenger runs the setup algorithms and gives the public parameters to $\mathcal {A}$.
3. The adversary may request attribute secret keys for arbitrary attributes and prover identifiers. For every identifier $I_u$, the set of attributes for which $\mathcal {A}$ obtains secret keys must not satisfy $\Gamma ^*$. The adversary may obtain keys associated with different identifiers; therefore, the experiment also captures attempts to combine attributes belonging to different provers.
4. The challenger generates a valid verification challenge for $\Gamma ^*$, including a uniformly selected challenge key $R\in G_T$ and nonce $r\in \mathbb {Z}_p$, and sends the challenge to $\mathcal {A}$.
5. The adversary outputs a response. It wins if the verifier accepts the response.

The soundness advantage of $\mathcal {A}$ is defined as

$$\textsf{Adv}^{\textsf{snd}}_{\mathcal {P},\mathcal {A}}(\lambda ) = \Pr \left[ \textsf{Exp}^{\textsf{snd}}_{\mathcal {P},\mathcal {A}}(\lambda )=1 \right] .$$

The protocol is sound if this advantage is negligible for every PPT adversary.

#### Definition 5

*(Attribute privacy)* In the attribute-privacy experiment, an adversarial verifier $\mathcal {A}$ selects an access policy *Γ* and two attribute vectors $AV_0$ and $AV_1$ such that

$$AV_0\models \Gamma \quad \text {and}\quad AV_1\models \Gamma .$$

The challenger selects a uniformly random bit *b*, executes the protocol using an honest prover possessing $AV_b$, and gives the resulting protocol view to $\mathcal {A}$. The verifier follows the specified challenge-generation algorithm while retaining the freedom to select the access policy. The adversary outputs a bit *b'*. Its advantage is

$$\textsf{Adv}^{\textsf{priv}}_{\mathcal {P},\mathcal {A}}(\lambda ) = \left| \Pr [b'=b]-\frac{1}{2} \right| .$$

The protocol provides attribute privacy if this advantage is negligible. This definition ensures that a verifier cannot distinguish which satisfying attribute vector was used. It does not hide the verification result itself. Across multiple verification sessions, the verifier obtains the result of each selected policy predicate, which is the intended information disclosed by the protocol.

#### Definition 6

*(Honest-verifier zero knowledge)* The protocol is honest-verifier zero knowledge if there exists a PPT simulator $\mathcal {S}$ that, given only the public parameters, the access policy *Γ*, and the fact that *Γ* is satisfied, produces a transcript that is computationally indistinguishable from the transcript of an execution between an honest prover and an honest verifier.

The simulator must not receive the prover’s attribute vector, attribute secret keys, or identifier.

#### Definition 7

*(Unlinkability)* Consider two protocol sessions generated either by the same honest prover or by two different honest provers whose attribute vectors satisfy the corresponding access policies. An adversary is given both protocol transcripts and outputs a bit indicating whether the transcripts were generated by the same prover.

The protocol is unlinkable if every PPT adversary distinguishes these two cases with only negligible advantage, provided that fresh challenge values and fresh encryption randomness are used in every session.

#### Definition 8

*(Untraceability)* In the untraceability experiment, an issuer issues the same type of attribute to two different honest provers. One of these provers subsequently participates in an attribute-verification session. The issuer is given the cryptographic transcript of that session and attempts to determine which prover used the issued attribute.

The protocol is untraceable if every PPT issuer succeeds with probability at most $1/2+\textsf{negl}(\lambda )$.

### Setup phases

#### Main setup

In the setup phase, the main parameters of the VeriPriv protocol are generated. The phase starts with the $global\_setup()$ algorithm, similar and based on DABE, which runs only once during the setup phase of the system and generates the global parameter $Q_a$. This parameter includes two cyclic groups, *G* and $G_T$, a generator *g* in *G*, a bilinear mapping $e:G\times G\rightarrow G_T$, a hash function $\mathcal {H}:\{0,1\}^*\rightarrow G$ that maps an identifier to an element in *G*, and a hash function $H:\{0,1\}^*\rightarrow \{0,1\}^d$ that maps any input to a *d*-bit digest. The groups *G* and $G_T$ have prime order *p*, and all scalar operations are performed over the finite field of *p* elements, with computations reduced modulo *p*. The global parameter is

$$\begin{aligned} Q_a=(G,G_T,g\in G,e(\cdot ,\cdot ),\mathcal {H}(\cdot ),H(\cdot )). \end{aligned}$$

Although the generation of the global bilinear-pairing parameters requires a one-time common setup at the beginning of the system lifecycle, all subsequent operations, including issuer setup, attribute-key issuance, challenge generation, and attribute verification, are performed in a decentralized manner without a central managing entity.

#### Issuer setup

The VeriPriv protocol allows organizations responsible for managing a given attribute *i* to join the system as independent issuers using the $attribute\_setup()$ algorithm at any time. To become an issuer, the organization chooses two random private keys, $\alpha _i$ and $\beta _i$, from the set of integers modulo *p*. These keys are kept private by the issuer, and the pair $e(g,g)^{\alpha _i}$ and $g^{\beta _i}$ is computed as its public key. This public key is then published as part of a public parameter $Q_b$, which includes the public keys of all issuers in the system. The structure of $Q_b$ is

$$Q_b=\big ((e(g,g)^{\alpha _0},g^{\beta _0}),(e(g,g)^{\alpha _1},g^{\beta _1}),\ldots \big ),$$

where $e(g,g)^{\alpha _i}$ and $g^{\beta _i}$ are the public-key components of the *i*-th issuer. An issuer can join the system at any time during the lifecycle of the protocol.

The setup is designed to provide a common algebraic domain for all participants while allowing each issuer to generate and manage its keys independently. The value $\alpha _i$ is used in the pairing-based component associated with attribute *i*, whereas $\beta _i$ binds the issued attribute secret key to the prover identifier through $\mathcal {H}(I_u)$. This binding is intended to prevent secret keys issued to different prover identifiers from being combined to satisfy a single access policy. Since each issuer selects $\alpha _i$ and $\beta _i$ independently, no coordination with other issuers is required after the one-time global setup.

#### Prover setup

In our system, each prover *u* is associated with a unique general identifier $I_u$ to prevent two malicious provers from combining their attributes to prove a challenge that they cannot solve independently. The identifier is globally unique for the prover, while its concrete form can depend on the application. The relevant issuers consistently use the same $I_u$ for that prover. The establishment and authentication of $I_u$ are provided by the surrounding application and are outside the attribute-verification protocol. To obtain the secret keys for its set of attributes, a participant contacts the relevant issuers and verifies its identity using its identifier $I_u$. An issuer of attribute *i* generates the corresponding secret key $sk_{(i,u)}$ by computing

$$sk_{(i,u)}=g^{\alpha _i}\mathcal {H}(I_u)^{\beta _i}.$$

### Verification modes

The verifier specifies the set of target attributes for verification $T_v=\{t_1,t_2,\ldots ,t_n\}$. Based on the verification requirement, the access policy *Γ* can be defined using the *1-out-of-n*, *n-out-of-n*, or *Combined Verification Mode*.

Let $AV_u$ be the attribute vector of prover *u*. The *1-out-of-n* and *n-out-of-n* verification modes are formally defined by the following predicates:

$$\Gamma _{\textrm{OR}}(AV_u,T_v)=1 \quad \Longleftrightarrow \quad AV_u\cap T_v\ne \varnothing ,$$

and

$$\Gamma _{\textrm{AND}}(AV_u,T_v)=1 \quad \Longleftrightarrow \quad T_v\subseteq AV_u.$$

For the *Combined Verification Mode*, let $\mathcal {T}_v=\{T_v^{(1)},T_v^{(2)},\ldots ,T_v^{(m)}\}$ be a collection of arbitrary non-empty groups of target attributes, and let $T_v=\bigcup _{j=1}^{m}T_v^{(j)}$ denote the complete target-attribute set. The corresponding predicate is

$$\Gamma _{\textrm{COMB}}(AV_u,\mathcal {T}_v)=1 \quad \Longleftrightarrow \quad \exists j\in \{1,\ldots ,m\}: T_v^{(j)}\subseteq AV_u.$$

Thus, the *1-out-of-n* mode accepts if the prover possesses at least one attribute in $T_v$, whereas the *n-out-of-n* mode accepts only if the prover possesses every attribute in $T_v$. The *Combined Verification Mode* accepts when the prover possesses all attributes of at least one group in $\mathcal {T}_v$. This mode therefore represents an OR of AND groups. Compared with the *n-out-of-n* mode, the verifier learns that one complete group satisfies the policy but does not learn which satisfying group was used from the cryptographic response. These predicates define only the verification result. The privacy guarantee is established separately by the attribute-privacy experiment and the simulation-based argument in the security analysis.

**n-out-of-n Verification:** In this mode, all attributes in $T_v$ must be satisfied to pass the verification. The access policy *Γ* is set to the logical AND of all attributes in $T_v$.

**1-out-of-n Verification:** In this mode, the verification passes when at least one attribute in $T_v$ is satisfied. The access policy *Γ* is set to the logical OR of all attributes in $T_v$.

**Combined Verification Mode:** In this mode, the target attributes are organized into non-empty groups. The attributes inside each group are connected by AND gates, while the groups are connected by an OR gate. Verification passes when the prover satisfies all attributes of at least one group.

### Attribute verification process

Let *Π =(Gen,Enc,Dec)* be a symmetric-key encryption scheme, where $Enc_k(m)$ and $Dec_k(m)$ denote the encryption and decryption of message *m* using symmetric key *k*, respectively. Let $\Pi _{\textrm{PKE}}=(KeyGen_{\textrm{PKE}},Enc_{\textrm{PKE}},Dec_{\textrm{PKE}})$ be a randomized public-key encryption scheme, where $Enc_{\textrm{PKE}}(PK_v,m)$ denotes encryption of message *m* under the verifier’s public key $PK_v$. It is used to protect the prover’s response. The concrete public-key encryption algorithm and the authentication of $PK_v$ depend on the surrounding application and are outside the current protocol.

#### Challenge phase

In the VeriPriv protocol, the verifier obtains the public keys of the intended issuers using the set of target attributes $T_v$. These public keys consist of the values $e(g,g)^{\alpha _i}$ and $g^{\beta _i}$. Let the corresponding LSSS representation be $(\mathcal {M}(\Gamma ),\rho )$, where $\mathcal {M}(\Gamma )\in \mathbb {Z}_p^{\ell \times q}$ and $\rho :\{1,\ldots ,\ell \}\rightarrow T_v$ maps each row to its corresponding attribute. Here, *ℓ* and *q* denote the numbers of rows and columns, respectively. The verifier generates two column vectors

$$\gamma = (s, \gamma_2, \dots, \gamma_q)^\top \in \mathbb{Z}_p^q$$

and

$$\omega = (0, \omega_2, \dots, \omega_q)^\top \in \mathbb{Z}_p^q,$$

where all components other than the first component of each vector are selected uniformly at random from $\mathbb {Z}_p$.

The verifier generates a random challenge key $R\in G_T$ and computes the symmetric key *H*(*R*). It also generates a fresh nonce $r\in \mathbb {Z}_p$ and records the challenge-generation timestamp *ts*. The nonce uniquely binds a prover’s response to the current challenge and prevents the reuse of a response from a previous protocol execution. The timestamp allows the verifier to determine whether the response was produced within the predefined freshness interval. The verifier encrypts the nonce, timestamp, and its public key as follows:

1 $$\begin{aligned} challenge=Enc_{H(R)}(r\mathbin {\Vert }ts\mathbin {\Vert }PK_v). \end{aligned}$$

To encrypt the randomly generated challenge key *R* in any verification mode, the verifier employs the DABE encryption scheme. If the verifier lacks the resources required to perform the encryption, it can use lightweight protocols such as SDABE [31], OEABE [32], or ODABE [33].

For each row *i* of $\mathcal {M}(\Gamma )$, the verifier selects a random scalar $r_i\in \mathbb {Z}_p$ and computes the scalar LSSS shares $\gamma _i,\omega _i\in \mathbb {Z}_p$ as follows:

2 $$\begin{aligned} \gamma _i=\mathcal {M}(\Gamma )_{i,*}\boldsymbol{\gamma }, \qquad \omega _i=\mathcal {M}(\Gamma )_{i,*}\boldsymbol{\omega }, \end{aligned}$$

where $\mathcal {M}(\Gamma )_{i,*}$ denotes the *i*-th row of the LSSS matrix. For the *1-out-of-n* mode, a simple representation is a single-column matrix with all entries equal to 1 and $\rho (i)=t_i$. In that case, $\gamma _i=s$ and $\omega _i=0$ for every row, so any one target row can reconstruct the shared value. The challenge key *R* is encrypted as

3 $$\begin{aligned} C_0=R e(g,g)^s. \end{aligned}$$

For each row *i*, the verifier computes the following three ciphertext components:

4 $$\begin{aligned} \begin{aligned} C_{i,1}&=e(g,g)^{\gamma _i}\left( e(g,g)^{\alpha _{\rho (i)}}\right) ^{r_i},\\ C_{i,2}&=g^{r_i},\\ C_{i,3}&=\left( g^{\beta _{\rho (i)}}\right) ^{r_i}g^{\omega _i}. \end{aligned} \end{aligned}$$

The challenge construction follows the secret-sharing structure of the access policy. The vector $\boldsymbol{\gamma }$ shares the secret value *s* among the rows of $\mathcal {M}(\Gamma )$, while $\boldsymbol{\omega }$ shares the value 0. Consequently, an authorized set of rows can reconstruct *s*, whereas the corresponding identifier-dependent terms reconstruct to zero and cancel during decryption. The random value $r_i$ independently randomizes the ciphertext components associated with each row. Finally, $C_0$ masks the challenge key *R* using $e(g,g)^s$. A prover whose attributes satisfy the access policy can reconstruct this masking value using the LSSS reconstruction coefficients and recover *R*; a prover whose attributes do not satisfy the policy cannot perform this reconstruction under the security of the underlying DABE scheme.

#### Response phase

The prover receives the challenge from the verifier and uses its attribute secret keys to construct the response. The response provides an honest-verifier zero-knowledge proof that the prover’s attributes satisfy the selected access policy, without revealing the attribute secret keys or additional attribute-dependent values in the cryptographic transcript. To solve the challenge, the prover uses its secret key $sk_{(\rho (i),u)}$ and the ciphertext tuple $C_i=(C_{i,1},C_{i,2},C_{i,3})$ associated with attribute *ρ (i)*. The prover calculates the following intermediate value:

5 $$\begin{aligned} \frac{C_{i,1}\,e(\mathcal {H}(I_u),C_{i,3})}{e(sk_{(\rho (i),u)},C_{i,2})} = e(g,g)^{\gamma _i}e(\mathcal {H}(I_u),g)^{\omega _i}. \end{aligned}$$

In the *1-out-of-n* verification mode, the prover must possess at least one attribute in the target set. For the OR-gate LSSS, the intermediate value corresponding to any possessed target attribute reconstructs the masking value as

6 $$\begin{aligned} e(g,g)^s=e(g,g)^{\gamma _i}. \end{aligned}$$

In the *n-out-of-n* verification mode, the prover must possess all attributes in the target set. In the *Combined Verification Mode*, the prover must possess all attributes of at least one target group. Let *I* denote the corresponding authorized set of rows of $\mathcal {M}(\Gamma )$. Since *I* satisfies the access policy, there exist reconstruction coefficients $c_i\in \mathbb {Z}_p$ such that

$$\sum _{i\in I}c_i\mathcal {M}(\Gamma )_{i,*}=(1,0,\ldots ,0).$$

Using the intermediate values computed in Eq. (5), the prover reconstructs $e(g,g)^s$ as follows:

7 $$\begin{aligned} \begin{aligned} e(g,g)^s = \prod _{i\in I} \left( e(g,g)^{\gamma _i}e(\mathcal {H}(I_u),g)^{\omega _i} \right) ^{c_i}. \end{aligned} \end{aligned}$$

The reconstruction property gives $\sum _{i\in I}c_i\gamma _i=s$ and $\sum _{i\in I}c_i\omega _i=0$, because the first components of $\boldsymbol{\gamma }$ and $\boldsymbol{\omega }$ are *s* and 0, respectively.

The prover recovers the challenge key *R* from $C_0$ using

8 $$\begin{aligned} R=\frac{C_0}{e(g,g)^s}. \end{aligned}$$

After recovering *R*, the prover decrypts the challenge:

9 $$\begin{aligned} Dec_{H(R)}(challenge)=r\mathbin {\Vert }ts\mathbin {\Vert }PK_v. \end{aligned}$$

Finally, the prover returns $Enc_{\textrm{PKE}}(PK_v,r\mathbin {\Vert }ts)$. Although the encrypted responses of different provers may differ because of the encryption randomness, the verifier decrypts each response and verifies that the recovered nonce is equal to the nonce stored for the corresponding challenge. The verifier also compares the recovered timestamp with its current local time and accepts the response only if the timestamp is within the predefined freshness interval. A nonce is accepted only once, preventing replay of a previously valid response.

**Fig. 3:**
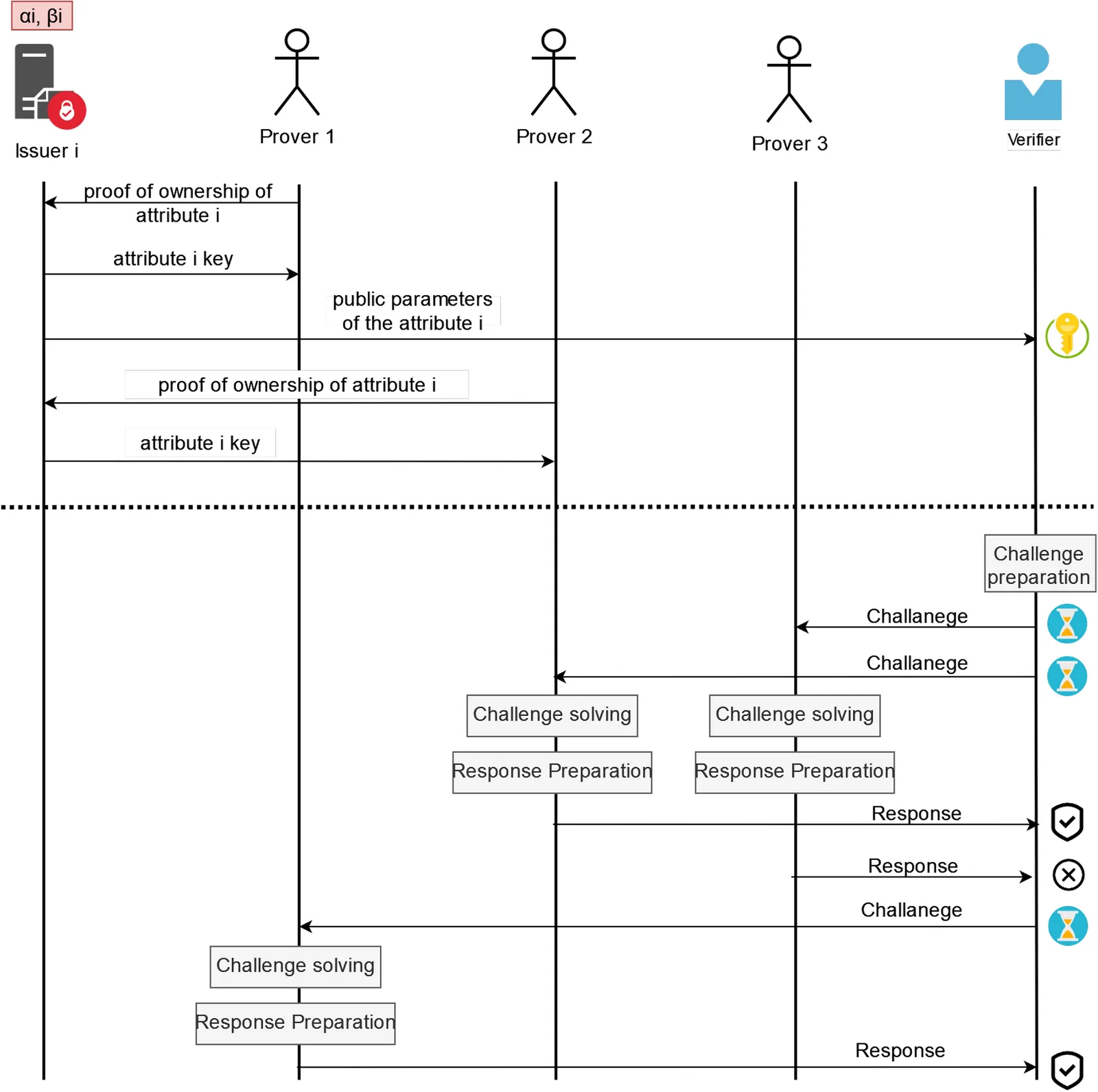
VeriPriv attribute verification protocol

Figure 3 illustrates the VeriPriv attribute verification protocol. The figure consists of an attribute issuer, three provers (two honest and one malicious), and a verifier on the rightmost side who intends to perform a *1-out-of-n* verification mode. The verifier specifies the set of target attributes for verification $T_v={t_0, t_1, \dots , t_n}$ and generates a random challenge key.

Next, the verifier selects a random number $s \in \mathbb {Z}_p$ and converts the access policy *Γ* into an equivalent LSSS matrix $\mathcal {M}(\Gamma )$. On the other hand, the provers can prove that their attributes satisfy the defined *Γ* when the corresponding predicate returns true.

## Evaluation

In this section, we evaluate the VeriPriv verification protocol through a preliminary computational benchmark, formal security analysis, and threat assessment. The computational evaluation measures the total protocol execution time with respect to the number of attributes in the Boolean access formula. The VeriPriv protocol has the following features:

1. Privacy-preserving verification: The protocol allows users to prove that their attributes satisfy the target access policy without revealing additional attribute-dependent values beyond the verification result.
2. Collusion-resistant verification: The underlying DABE construction prevents provers from combining keys associated with different identifiers to satisfy a challenge that none can satisfy independently.
3. Decentralized verification: After the one-time global setup, the protocol does not rely on a single managing entity for attribute issuance or verification. Its scalability with respect to the number of participants requires further empirical evaluation.

### Computational evaluation

The protocol was implemented in Python using the Charm cryptographic framework. The implementation was evaluated on a computer equipped with an 8th-generation Intel Core i7 U-series processor operating at 2.1 GHz with turbo frequencies up to 3.9 GHz, and 16 GB DDR4-2400 SDRAM. The symmetric encryption layer was instantiated using AES-GCM. Each evaluated configuration was executed 20 times, and Table 1 shows the average total protocol execution time for Boolean formulas containing two, three, and four attributes. The corresponding average execution times were 43, 58, and 73 milliseconds, respectively. The results indicate that the total execution time increases as the number of attributes in the Boolean formula increases.

**Table 1:** Execution time for different numbers of attributes in the Boolean formula

Number of attributes	Execution time (ms)
2	43
3	58
4	73

This preliminary evaluation provides an initial indication of the computational cost of the implemented protocol on the evaluated general-purpose computer. The values measure the total protocol execution time. Evaluation on representative resource-constrained IoT devices, together with communication, memory, and storage measurements, remains part of future experimental work.

### Security analysis

#### Theorem 1

(Soundness) Assume that the underlying DABE scheme is collusion resistant and IND-CPA secure under its stated assumptions, *H* is a secure key-derivation function, and *Π* is IND-CPA secure. Then the VeriPriv protocol satisfies the soundness definition.

#### Proof

Let $\mathcal {A}$ be a malicious prover that does not possess a set of attribute secret keys satisfying the target policy $\Gamma ^*$. We consider a sequence of games.

In the first game, the challenger performs the real soundness experiment. To construct an accepted response, $\mathcal {A}$ must return the nonce *r* contained in

$$Enc_{H(R)}(r\mathbin {\Vert }ts\mathbin {\Vert }PK_v).$$

In the second game, the challenge key encrypted in the DABE ciphertext is replaced by an independent random value from $G_T$. An adversary that distinguishes the first and second games can be used to distinguish two DABE plaintexts without possessing an authorized set of attribute keys. The difference between these games is therefore bounded by the IND-CPA advantage against the underlying DABE scheme.

The key *H*(*R*) is then replaced by an independently selected symmetric key. The difference is bounded by the security of the key-derivation function *H*. Under the IND-CPA security of *Π*, the encrypted challenge reveals no computationally useful information about the nonce *r*.

Consequently, an adversary that has not decrypted the challenge can produce an accepted response only by guessing *r*. Since *r* is selected uniformly from $\mathbb {Z}_p$, this event has probability 1/*p*. Hence, for suitable PPT adversaries $\mathcal {B}_1$, $\mathcal {B}_2$, and $\mathcal {B}_3$,

10 $$\begin{aligned} \begin{aligned} \textsf{Adv}^{\textsf{snd}}_{\mathcal {P},\mathcal {A}}(\lambda ) \le {}&\textsf{Adv}^{\mathsf {ind\text{- }cpa}}_{\textsf{DABE},\mathcal {B}_1}(\lambda ) + \textsf{Adv}^{\textsf{kdf}}_{H,\mathcal {B}_2}(\lambda )\\&+ \textsf{Adv}^{\mathsf {ind\text{- }cpa}}_{\Pi ,\mathcal {B}_3}(\lambda ) + \frac{1}{p}. \end{aligned} \end{aligned}$$

All terms are negligible under the stated assumptions. The restriction that no set of keys associated with a single identifier satisfies $\Gamma ^*$, together with the collusion resistance of the DABE scheme, also prevents attributes belonging to different provers from being combined successfully.

For the selected instantiation, the first advantage term is bounded by the established security reduction of that DABE construction under its stated assumptions. If another DABE instantiation is used, the corresponding underlying security assumption applies through the same protocol-level reduction. *□*

#### Theorem 2

(Attribute privacy and honest-verifier zero knowledge) The VeriPriv protocol satisfies attribute privacy and honest-verifier zero knowledge with respect to the cryptographic transcript.

#### Proof

For any attribute vector satisfying *Γ*, the prover’s response contains only the nonce *r* and timestamp *ts*, encrypted under $PK_v$ using randomized public-key encryption. Neither value depends on the particular attributes or secret keys used to recover *R*.

Therefore, two satisfying attribute vectors produce identically distributed response plaintexts. The verifier learns only that the policy is satisfied and obtains no additional attribute-dependent value from the protocol.

A simulator can generate an honest-verifier transcript without knowledge of the prover’s attributes. It selects *R*, *r*, *s*, *ts*, and the required encryption randomness, constructs the DABE challenge using the public attribute keys, computes

$$Enc_{H(R)}(r\mathbin {\Vert }ts\mathbin {\Vert }PK_v),$$

and encrypts the corresponding response under $PK_v$ using the public-key encryption algorithm. The resulting distribution is identical to that of an honest execution. Thus, the protocol is honest-verifier zero knowledge and provides attribute privacy. *□*

#### Theorem 3

(Unlinkability and untraceability) Assume that every verification session uses fresh challenge values and fresh encryption randomness. Then the cryptographic transcripts of the protocol satisfy unlinkability and untraceability.

#### Proof

The messages returned by a prover do not contain its identifier, its attribute secret keys, or any deterministic value derived from either of them. The returned plaintext consists only of fresh verifier-generated values. Therefore, conditioned on the same public verification result, the transcript distribution is independent of the identity of the prover.

It follows that transcripts produced by the same prover and transcripts produced by different provers have the same distribution in the stated model. Hence, a verifier cannot link two sessions using only the cryptographic transcripts.

The issuer participates in attribute issuance but does not contribute any prover-specific value to the later challenge–response exchange. Consequently, the issuer cannot associate a verification transcript with one of the users to whom it issued an attribute, except with probability $1/2+\textsf{negl}(\lambda )$. *□*

### Threat assessment

VeriPriv protocol is designed to protect against a range of potential threats and adversaries, including:

- Malicious Users: Users with the intent to impersonate others or gain unauthorised access.
- Insider Threats: Authorized participants who may attempt to misuse their roles or protocol information.
- Eavesdroppers: Attackers attempting to intercept communication between users and the verification system.
- Attribute Manipulation: Adversaries trying to use forged, invalid, or improperly combined attribute keys during verification.

In the VeriPriv protocol for attribute verification, several cryptographic and system measures are combined to address these threats. The model assumes that issuers correctly validate an attribute before issuing its corresponding secret key. The authenticity of issuer public keys in $Q_b$ is provided by the surrounding deployment environment.

#### Malicious users

To mitigate the risk posed by malicious users, the VeriPriv protocol requires the prover to recover the challenge key using a set of valid attribute secret keys that satisfies the selected access policy. The soundness property prevents a prover that does not possess a satisfying set of keys from producing an accepted response except with negligible probability. The identifier binding in the underlying DABE construction also prevents colluding provers from combining attribute keys issued to different identifiers to satisfy a policy that none of them can satisfy independently.

#### Insider threats

The VeriPriv protocol separates the participants into the roles of issuer, verifier, and prover. Issuers are responsible for validating attributes and issuing the corresponding keys, verifiers define the access policy and generate protocol-conforming challenges, and provers use their issued keys to satisfy the selected policy. This separation limits each role to the information and operations required for its function. The attribute-privacy and untraceability properties further limit the attribute-dependent information available from later verification transcripts. Secure key exchange mechanisms can be integrated to protect communication between the issuer and prover during attribute-key issuance.

#### Eavesdroppers

To mitigate the risks posed by eavesdroppers, the challenge content is protected using the symmetric key derived from *R*, and the prover’s response is encrypted under the verifier’s public key. The attribute secret keys and the prover identifier are not transmitted in the verification response. Therefore, an eavesdropper observing the cryptographic messages cannot directly recover the protected challenge values or the prover’s attribute secret keys under the stated encryption assumptions.

#### Attribute manipulation

The soundness and collusion-resistance properties provide the main protection against manipulation of attribute proofs. A prover can pass verification only when its valid attribute keys satisfy the selected policy, while keys issued to different prover identifiers cannot be combined successfully. Attribute privacy has a separate role: it limits disclosure of the prover’s attribute vector beyond the result of the selected access-policy predicate. Together, these properties maintain the integrity and privacy of the attribute-verification process.

By incorporating these measures, the VeriPriv protocol addresses malicious users, insider misuse within the stated role assumptions, eavesdropping, and attribute manipulation while preserving privacy during attribute verification.

## Applications

The VeriPriv protocol for attribute verification can be utilized in various research domains. In this section, some of the applications and use-cases of the VeriPriv protocol are briefly mentioned.

### Internet of things

During resource discovery in IoT networks, the clients have to reveal some of their attributes during the process of resource discovery [34]. Although it is a necessary requirement, it leads to privacy issues [35]. The attributes of the clients in some resource discovery protocols can be hold by their directly connected gateways and not included in the discovery request. Although it partially address the privacy concern, but it causes efficiency problems [36]. To address this requirement without revealing the actual attributes of the client, *1-out-of-n* verification mode can be used to check that the issuer client has one of the required attributes, without revealing it. Additionally, different machines in Industrial Internet of Things(IIoT) have different authentication mechanisms. Generally, in some cases, a node needs to assure a specific level of authentication. As instance, in multi factor authentication, any authentication factor would be sufficient to authenticate the machine. To do so, without revealing the actual credentials for authentication, *1-out-of-n* verification mode can be used.

### Industrial internet of things

The VeriPriv protocol for attribute verification can be effectively utilised in the domain of IIoT as an authorization component for secure data sharing and secure device-update mechanisms. The strict security requirements in IIoT, such as maintaining data confidentiality, integrity, and authenticity, make it crucial to establish robust mechanisms for secure communication and access control. In IIoT environments, multiple devices and systems need to exchange sensitive data while ensuring its confidentiality and integrity. The VeriPriv protocol provides a framework for verifying the attributes of users and devices involved in data sharing. By utilising zero-knowledge proofs and cryptographic techniques, the protocol ensures that only authorised and authenticated entities can access and share data. The sensitive data can be encrypted and decrypted using the cryptographic keys issued through the protocol, thus preventing unauthorised data exposure. The attribute verification process enhances the overall security of data sharing in IIoT, minimising the risk of data breaches and unauthorised access.

Maintaining the security and integrity of devices and their software is crucial in IIoT systems. The VeriPriv protocol can be integrated as an authorization component of a secure device-update mechanism. By incorporating attribute verification during the update process, the update system can restrict update access to devices possessing the required attributes. The remaining update-security functions, such as update authenticity and integrity verification, can be provided by the update mechanism itself. In this way, attribute verification provides an additional authorization layer for IIoT device management.

### Federated learning

FL [37] provides a new machine learning paradigm by training the data on the local datasets and aggregating updated models from participants on the server, which was first proposed by McMahan et al. [38]. The technology gains popularity as it can offer some level of data security and privacy protection [39]. Recent FL research has begun to investigate stronger mechanisms for participant authentication and authorization. SecureFedPROM [40] integrates attribute-based access control into a zero-trust federated-learning framework for client authorization, while a recent blockchain-based FL framework uses decentralized identifiers and verifiable credentials to authenticate participating entities [41]. With these attribute verification modes, we can verify the attributes of clients. From the privacy point of view, the protocol authenticates clients based on attributes while limiting disclosure of the underlying attribute information. Grouping clients according to verified attributes may also support the selection of participants with similar properties, which can be useful for faster convergence or personalized federated learning depending on the learning task and deployment.

### Data validation

The data validation networks such as D3VN [42] consists of various nodes distributed in different geographical locations. These validation protocols use the existing processing capacities in the network, e.g. unused computers in production environments or rarely used servers for the validation of data. As any node can join the network, the joined data validators in such protocols can be malicious. Therefore, the selection process involves the attributes of the data validators in a decentralized approach, to mitigate the affect of malicious data validators [30]. Since the selection of the data validators is done based on the attributes of those validators, it calls into question the legitimacy of the claimed attributes of the data validators. The *n-out-of-n* verification mode can be used to prove the the legitimacy of the claimed attributes of the involved data validators.

### Access control

Access control is a vital component of many systems to protect sensitive resources and prevent unauthorised access. The VeriPriv protocol strengthens access control mechanisms by verifying the attributes of users and devices before granting access to critical resources. Only users or devices possessing the required attributes can successfully pass the attribute verification process and gain access to specific resources or functionalities. This attribute-based access control model provides fine-grained control over who can access what resources within the network, minimising the risk of unauthorised access and potential security breaches.

By utilising the VeriPriv protocol for attribute verification, IIoT systems can strengthen authorization for data sharing, device-update mechanisms, and access control. The protocol verifies whether users or devices satisfy the required attribute policies before access is granted, while the surrounding IIoT system provides the additional confidentiality, integrity, and update-security mechanisms required by the application. Recent decentralized attribute-based access-control research [27] has shown that participating domains can retain independent policies and attribute authorities while supporting privacy-preserving cross-domain access decisions.

### B5G authentication

Recent work on Beyond 5GB5G and 6G networks has highlighted an authentication challenge during the early access phase, where a User Equipment(UE) may exchange and trigger signaling before the standard authentication procedures in the core network are completed. In such a setting, unauthorized or malicious devices may consume access and core-network resources before their legitimacy is verified. An edge-assisted pre-authentication approach has therefore been proposed [43] to allow the Next Generation Node B(gNB) to perform an early trust decision based on selected UE attributes before forwarding further signaling toward the core network. The VeriPriv protocol can be a possible fit for this authentication challenge. The gNB can act as the verifier and define a set of attributes required for the early trust decision, while the UE acts as the prover. Using the proposed protocol, the UE can prove that its attribute vector satisfies the required access policy without revealing its complete set of attributes.

The different verification modes can support different pre-authentication requirements. The *1-out-of-n* mode can be used when any one of several accepted attributes is sufficient for the initial trust decision. The *n-out-of-n* mode can be used when all required attributes must be satisfied. The Combined Verification Mode can also be applied when multiple acceptable groups of attributes are defined, where the UE needs to satisfy all attributes in one of the groups without revealing which group has been used.

## Conclusion

In this paper, we proposed VeriPriv, an attribute verification protocol with *1-out-of-n*, *n-out-of-n*, and *Combined Verification Mode*. In *1-out-of-n* verification mode, the verifier is able to verify that a specific prover has one of the required attributes, without learning which attribute was used to satisfy the policy. In *n-out-of-n* verification mode, the verifier is able to verify that a specific prover has all the required attributes. In the *Combined Verification Mode*, the verifier can define multiple non-empty groups of attributes connected by OR, while the attributes inside each group are connected by AND. The verifier learns that the prover satisfies at least one complete group without learning which satisfying group was used from the cryptographic response. The VeriPriv attribute verification protocol verifies policy satisfaction in a decentralized and honest-verifier zero-knowledge approach with respect to the cryptographic transcript. The security analysis establishes soundness, attribute privacy, honest-verifier zero knowledge, unlinkability, and untraceability under the stated assumptions.

Despite its features, there are some open problems regarding the VeriPriv protocol. Although the issuers can be established independently, the initial setup requires that the global parameters including the groups for bilinear operations and the generators be computed. Additionally, immediate revocation of attributes and compromised attribute keys is not considered in the current version of VeriPriv and requires an additional revocation mechanism. A possible approach for attribute expiration is to allow issuers to issue secret keys for a defined validity period, after which a new key must be obtained. For instance, instead of a general studentship attribute issued by a university, the university can issue a studentship attribute that is joined with a specific academic year. However, this results in a larger number of attributes. Although a preliminary computational evaluation has been provided, future work should evaluate the protocol on representative Internet of Things(IoT) devices and measure its communication overhead, memory use, and storage requirements.

## Data Availability

No datasets were generated or analysed during the current study.
